# Tailor-Made Nanoporous Metal–Organic Frameworks Via Molecular Engineering for Bilirubin-Specific Capture from Liver Failure Patients’ Blood

**DOI:** 10.1007/s40820-026-02337-y

**Published:** 2026-09-16

**Authors:** Shanshan Zhang, Jinming Zhang, Yihan Li, Dabao Shang, Ding Zhao, Fangke Zhang, Juan Wang, Liang Chen, Xiaogang Xiang, Wenguo Cui

**Affiliations:** 1 Department of Orthopaedics, Shanghai Key Laboratory for Prevention and Treatment of Bone and Joint Diseases, Ruijin Hospital, Shanghai Institute of Traumatology and Orthopaedics, Shanghai Jiao Tong University School of Medicine, 197 Ruijin 2Nd Road, Shanghai, 200025 People’s Republic of China; 2 School of Nursing, Shanghai Jiao Tong University, 227 South Chongqing Road, Shanghai, 200025 People’s Republic of China; 3 Department of Infectious Diseases, Translational Laboratory of Liver Diseases, Ruijin Hospital, Shanghai Jiao Tong University School of Medicine, 197 Ruijin 2Nd Road, Shanghai, 200025 People’s Republic of China

**Keywords:** Molecule engineering, Metal–organic frameworks, Nanocrystals, Specific bilirubin capture, Blood purification

## Abstract

Overcoming the albumin–bilirubin binding barrier to achieve specific recognition and high-efficiency capture of blood bilirubin remains a major clinical challenge in treating patients with hyperbilirubinemia due to liver failure, cholestasis, or other related conditions. Here, a molecular engineering-assisted functionalization strategy is proposed: programming the coordination environment of nanocrystalline frameworks with monodentate carboxylic acid ligands of varied carbon chain lengths to construct a family of zirconium-based UiO-66 nanoporous frameworks that act as bilirubin-specific molecular capture traps. The experimental results and molecular dynamics simulations confirm that the newly engineered UiO-66 nanoporous framework rapidly enriched albumin-bound bilirubin through short-range van der Waals/electrostatic effects; the internal Zr–μ_3_–OH sites then formed directional Zr–pyrrole coordination with the bilirubin tetrapyrrole core, whereas albumin was physically excluded by the narrow apertures of the engineered nanoporous framework (average 7.6 Å), breaking the “albumin barrier” without competitive displacement. Dense intrapore hydrogen-bonding networks and π–π stacking further strengthen bilirubin-specific complexation. The computed adsorption energy (ΔE_ads_ = – 3.7 eV) indicates spontaneous, diffusion-limited capture. In simulated blood experiments, the newly developed UiO-66 nanoporous framework achieves an ultrahigh bilirubin removal rate of ~ 97.9% with negligible albumin loss. Finally, clinical tests on plasma samples from ten liver failure patients revealed a 251% higher bilirubin removal capacity per unit mass than that of the commercial ion exchange resin (IER) within 3 h, while albumin leakage remained ≤ 2.58% (vs. ≥ 8.53% for commercial IER). This study reveals, for the first time, the advantages and translational prospects of molecularly engineered nanocrystalline frameworks as a clinically viable platform for high-efficiency blood bilirubin removal.

## Hightlights

- This work innovatively formulated a molecular engineering strategy for nanoframeworks, achieving precise customization of their nanoporous architectures.
- This work developed a nanoscale bilirubin-specific capture trap, physically overcoming the critical trade-off between bilirubin removal and protein loss.
- A groundbreaking 251% increase in total bilirubin capture capacity, coupled with an albumin loss rate of ≤2.58%, established a new world clinical assessment record.

## Introduction

Hyperbilirubinemia can arise from various etiologies, including liver failure and cholestasis. Severe hyperbilirubinemia may further compromise hepatic synthesis, detoxification, and metabolic functions, ultimately leading to multiorgan dysfunction. Clinically, it manifests as jaundice, coagulopathy, hepatorenal syndrome, hepatic encephalopathy, and ascites [1–3]. Globally, liver diseases associated with severe hyperbilirubinemia, such as liver failure, affect millions of patients and account for approximately 2 million deaths annually [4]. Short-term mortality rates range from 40% to 70%, depending on the underlying etiology and the availability of regional healthcare resources [5, 6]. Plasma absorption-based artificial liver support systems play important supportive roles in treating patients with hyperbilirubinemia; however, their nonselectivity and transient efficacy have limited their large-scale application in clinical practice. Currently, the resin-based positive charge absorption process is largely nonselective, resulting in the unintended removal of beneficial plasma components such as albumin (Alb), immunoglobulins, complement proteins, and coagulation factors, which may exacerbate hypoalbuminemia, impair immune defense, and increase the risk of infection or bleeding [7]. This limitation stems from the nanomolar affinity (K_d_ ≈ 10^−8^ M) between bilirubin and Alb (69 kDa, hydrated diameter > 6 nm), which results in the formation of a kinetically stable complex with a dissociation half-life of 6–8 ms at physiological pH [8–10]. Consequently, existing sorbents clear only 20%–40% of free bilirubin and are virtually ineffective against the Alb-bound fraction [11–13]. Therefore, adsorbents that selectively capture total bilirubin (TBIL) while sparing essential plasma proteins are urgently needed. However, conventional materials are hampered by low-density, weak-affinity recognition sites, high in situ dissociation energy barriers and monotonous pore architectures, precluding selective fractionation under high-throughput conditions [14–17]. To address this, an integrated “recognition–dissociation–capture” platform is urgently needed for exclusive and rapid TBIL removal at physiological flow rates, thus markedly increasing purification efficiency.

Metal–organic frameworks (MOFs), a quintessential class of nanocrystalline materials [18], offer unmatched advantages in terms of molecular recognition and separation owing to their designable topologies [19–21], hierarchical porosity [22, 23], and dense active sites [24–26]. Ligand pre-functionalization or post-synthetic modification allows the directional installation of high-affinity motifs without compromising crystalline integrity, enabling exclusive guest capture [27–29]. Representative examples include urease-functionalized MOF-808-U, oxalate-modified MOF-808-OA, and Cu^2+^-doped MOF-808-Cu for urinary urea recovery [30], as well as a tryptophan–histidine-bifunctionalized Cu-MOF that achieves nanomolar detection of ascorbic acid in sweat [31]. These studies demonstrate that molecularly encoding the coordination environment of robust platforms such as zirconium-based UiO-66 nanocrystals [32, 33] can precisely match the geometric, electronic, and energetic demands of a target molecule [34–36], overcoming conventional limits in affinity, selectivity, and kinetics [37–39]. Consequently, engineering the coordination sphere of nanocrystalline UiO-66 through molecular programming represents a promising route toward the development of MOF-based sorbents specifically tailored for TBIL removal.

**Fig. 1:**
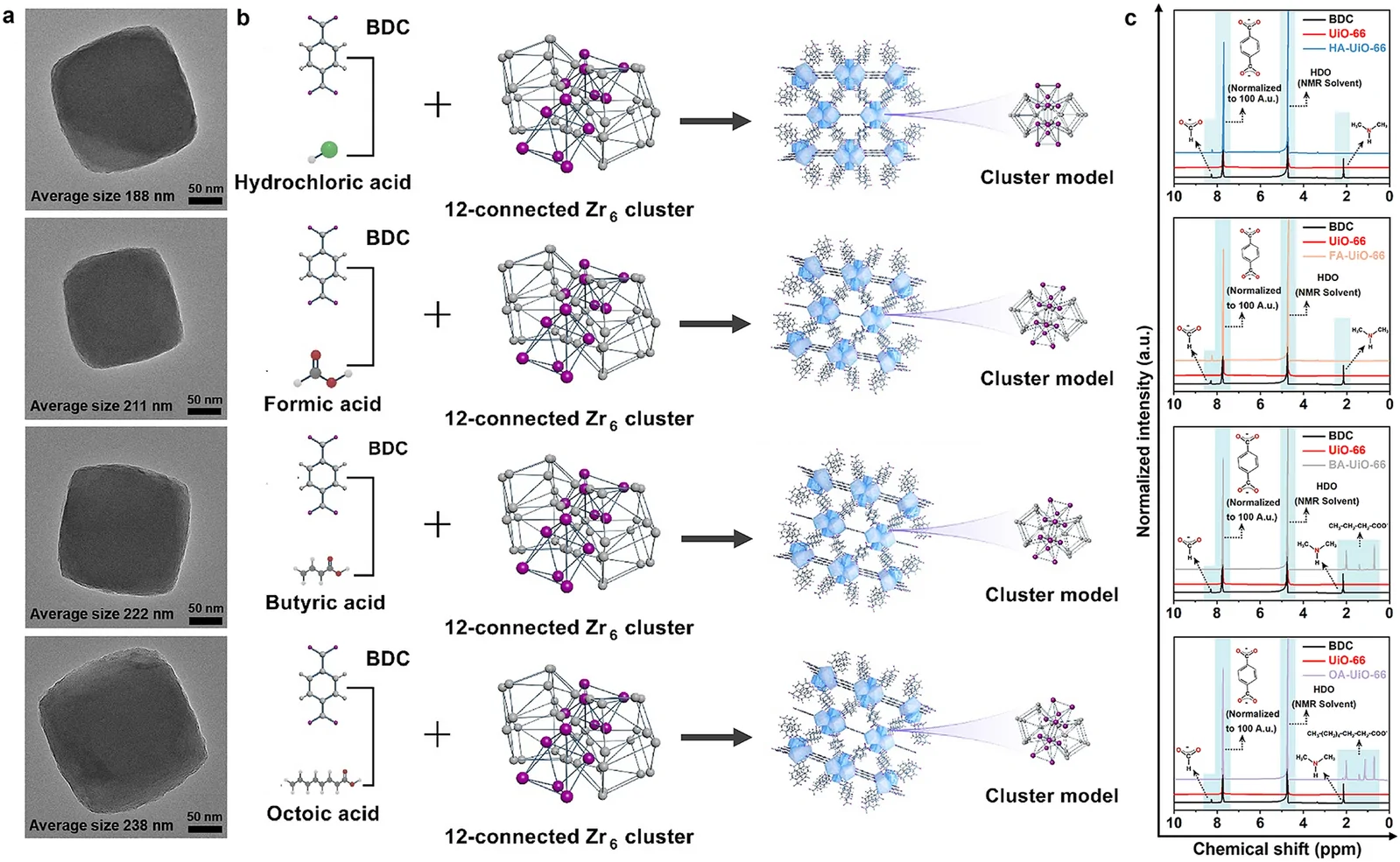
**a** TEM images,** b** chemical synthesis process, and **c** ^1^H NMR spectra of engineered nano-HA-UiO-66, FA-UiO-66, BA-UiO-66, and OA-UiO-66, respectively

Drawing inspiration from these precedents, we conceptualize an unusual strategy—“ligand-engineered programmable nanocrystalline MOFs”—and demonstrate their first application for blood purification in patients with liver failure as bilirubin-specific molecular capture traps. Monodentate carboxylic acids with increasing alkyl chain length (hydrochloric acid (HA), formic acid (FA), butyric acid (BA), and octanoic acid (OA)), uniformly recorded as XA, high p*K*a) are employed as minimal code units to competitively displace some of the pre-coordinated BDC ligands (low p*K*a) on Zr–O clusters, yielding a family of zirconium-based UiO-66 nanoporous framework variants under hydrothermal conditions (Fig. 1). Subsequent thermal activation removes residual XA and releases lattice strain, producing Zr–μ_3_-OH-rich bilirubin traps featuring three synergistic advantages: (i) Zr–μ_3_-OH forms endocyclic metal–pyrrole coordination with bilirubin tetrapyrrole, disrupting the electrostatic/van der Waals interactions within the bilirubin–Alb complex [40]; (ii) the preexisting local strain field supplies energy for in situ dissociation of the complex; and (iii) an ~ 7.6 Å pore aperture, reinforced by π–π stacking and hydrogen bonding, enables transient confinement of free bilirubin. This work closes the loop between “clinical need, chemical design, and material implementation” and offers an immediately translatable, scalable platform for selective TBIL removal in blood purification.

## Experimental Section

### Materials

Zirconium tetrachloride (ZrCl_4_, Purity 98%), terephthalic acid (H_2_BDC, Purity ≥ 98%), formic acid (FA, Purity ≥ 97%), n-butyric acid (BA, Purity ≥ 99%), n-octanoic acid (OA, Purity ≥ 99%), hydrochloric acid (HA, Purity ≥ 30%), N,N-dimethylformamide (DMF, AR), cell-grade dimethyl sulfoxide (DMSO) and anhydrous methanol (AR, Purity ≥ 99.9%) were purchased from Macklin (Shanghai, China). Bilirubin (Purity 99%), bovine serum albumin (BSA), urea, glucose and other bioactive components were obtained from Aladdin (Shanghai, China) and stored at –20 °C. Commercial ion exchange resin (IER) bilirubin adsorption product was supplied by a biotechnology company (Zhuhai, China). All the chemicals were used as received without further purification.

### Methods

#### Synthesis of the Engineered UiO-66 Nanoporous Frameworks

*Solution A*: ZrCl_4_ (0.466 g, 2 mmol) was dissolved in DMF (30 mL) and the desired monodentate carboxylic acid (HA, FA, BA or OA) was added to give a ZrCl_4_: linker: XA molar ratio of 1: 0.5: 25. The mixture was stirred magnetically at 80 °C for 30 min until complete dissolution occurred. *Solution B*: H_2_BDC (0.162 g, 1 mmol) was dissolved in DMF (30 mL) under the same conditions. Solutions A and B were combined, sonicated for 10 min, transferred to a Teflon-lined autoclave, degassed, and heated at 120 °C for 24 h. After natural cooling, the white solid was collected by centrifugation (10,000 rpm, 10 min) and purified as follows: (i) immersion in 1.25 vol% HCl/DMF (160 mL, 12 h, 2 ×) to remove residual acid/salts; (ii) washing with DMF (3 ×) and methanol (3 ×), centrifuging each time at 8000 rpm (10 min); and (iii) solvent exchange with methanol (2 ×24 h) to displace high-boiling DMF [41, 42]. The powders were dried under vacuum at 75 °C for 12 h to yield the engineered UiO-66 nanoporous frameworks, denoted as nano-HA-UiO-66, FA-UiO-66, BA-UiO-66, or OA-UiO-66 according to the acid used and stored in a desiccator for further use.

#### Characterization

Nanocrystalline structures were determined with X-ray diffraction (XRD, Cu-Kα radiation, 5–90° 2*θ* range, 0.02° step width, SmartLab SE, Rigaku, Japan). Morphologies were imaged using field-emission scanning electron microscopy (SEM, Sigma, ZEISS, Germany) and high-resolution transmission electron microscopy (HRTEM, 300 kV accelerating voltage; JEOL-2100F, Japan). The fine microstructure was characterized using an aberration-corrected transmission electron microscope (AC-TEM, model JEM-ARM300F, Japan). Surface functionalities were probed by Fourier transform infrared (FT-IR, Nicolet iS20, Thermo Fisher Scientific, USA) and X-ray photoelectron spectroscopies (XPS, K-Alpha, Thermo Scientific, USA), and binding energies were calibrated to C 1*s* at 284.8 eV. The fluorescence intensity of the as-synthesized materials was recorded on a fluorescence spectrometer (F-7100, Hitachi, Japan) under 280 nm excitation. N_2_ adsorption‒desorption isotherms (77 K) were measured with an Autosorb IQ instrument (Quantachrome, USA); specific surface areas were calculated using the Brunauer–Emmett–Teller (BET) equation, and pore size distributions were derived from the Barrett–Joyner–Halenda (BJH) model. The particle size distributions and ζ-potentials were obtained with a laser diffraction particle analyzer and an electrophoretic light-scattering system (SurPASS 3, Anton Paar, Austria). Zirconium leaching was quantified by inductively coupled plasma–optical emission spectroscopy (ICP‒OES, 5110, Agilent, USA). Carbon, hydrogen, and nitrogen contents were determined with an elemental analyzer (Vario EL cube, Elementar, Germany). Oxygen vacancy signals were detected by electron paramagnetic resonance (EPR, Bruker, Germany). Thermogravimetric and differential scanning calorimetry (TGA/DSC) analyses were performed on a Netzsch STA 449 F1 Jupiter (Germany) from 30 to 800 °C at 10 °C min^−1^ in air, with a 30 min isothermal hold at 800 °C. The removal of capping agents was verified by ^1^H NMR (400 MHz, Bruker, Germany). The role of defect structures in bilirubin adsorption was evaluated by X-ray absorption spectroscopy (XAS).

### Performance Testing

#### Phosphoric Acid (PA) Competitive Occupation of the Zr–μ₃-OH Site

To prepare phosphate‑occupied HA-UiO‑66 (denoted as PA‑UiO‑66), 100 mg of HA‑UiO‑66 powder was immersed in 20 mL of 0.1 M Na_2_HPO_4_ aqueous solution. The suspension was shaken at room temperature (25 ± 2 °C) for 12 h to ensure that the Zr–μ_3_-OH sites achieved sufficient coordination equilibrium. The sample powder was then collected by centrifugation (10,000 rpm, 5 min), washed three times with deionized water, and dried under vacuum at 60 °C for 12 h. The resulting PA‑UiO‑66 was stored in a vacuum desiccator until further use.

#### Bilirubin Capture Simulation Experiment

PBS (pH ≈ 7.4) was used to mimic physiological blood conditions. Bilirubin powder was first dissolved in a minimal volume of DMSO and 0.1 M Na_2_CO_3_ and then stepwise diluted with PBS to the required concentration to prevent precipitation. All manipulations were performed in the dark at 37 °C to avoid photodegradation. Each experiment was run in triplicate under identical conditions.The standard reference values for the TBIL and albumin concentrations in the simulated blood are provided in Table S1. Kinetic adsorption tests were conducted by dispersing 100 mg of the developed UiO-66 nanoporous frameworks in 50 mL of simulated blood (150 mg L^−1^ TBIL and 50 g L^−1^ BSA) at 37 °C under orbital shaking (200 rpm). At predetermined intervals, 1 mL aliquots were withdrawn, filtered through 0.45 µm membranes, and centrifuged (5 min, 10,000 rpm). The supernatant was analyzed for the TBIL concentration. The adsorption capacity (q) and removal efficiency (R, %) were calculated using Eqs. (1) and (2):

1 $$q\,(mg/g) = \frac{{(C_{0} - C_{e} ) \times V}}{W}$$

2 $$R\,(\% ) = \frac{{C_{0} - C_{e} }}{{C_{0} }} \times 100\%$$

Here, *C*_*0*_ and *Ce* (mg L^−1^) represent the initial and equilibrium concentrations of TBIL in solution, respectively; *V* (L) is the solution volume; and *W* (g) is the mass of the developed UiO-66 nanoporous frameworks.

#### Hemolysis Testing

Male Sprague–Dawley (SD) rats (age: 7–9 weeks; body weight: 200–250 g) were purchased from Beijing Vital River Laboratory Animal Technology Co., Ltd. The rats were housed in a specific pathogen-free (SPF) barrier facility under controlled conditions (temperature 22 ± 2 °C, relative humidity 50%–60%, 12 h light/12 h dark cycle) with free access to food and water. After one week of acclimatization, blood samples were collected for hemolysis assays. All animal protocols were approved by the *Institutional Animal Care and Use Committees of Shanghai Jiao Tong University School of Medicine* (IACUC-20240301–01) and Shanghai Pintai Biotechnology Co., Ltd. (AUP-20250516–02), and the experiments were performed in strict accordance with the relevant guidelines.

*Preparation of animal blood samples* Fresh whole blood was collected from healthy rats and immediately mixed with 3.8% sodium citrate (v/v, 1:9) to yield an anticoagulated sample. Platelet-poor plasma was obtained by centrifugation at 4000 rpm for 15 min and stored at 4 °C until use.

*Hemolysis assay* The engineered UiO-66 nanoporous framework powders were incubated with rat erythrocytes at 37 °C for 1 h to evaluate their hemolytic potential. Whole blood (1 mL) was centrifuged at 3,000 rpm for 10 min; the supernatant was discarded and the erythrocyte pellet was washed three times with PBS and resuspended in 10 mL of the same buffer. After pre-washing with PBS, graded amounts of nanocrystalline powders (1.0, 3.0, 5.0, 10.0, and 20.0 mg) were added to the suspension. Deionized water and PBS served as positive and negative controls, respectively. Following incubation at 37 °C for 1 h, the mixtures were centrifuged at 3000 r min^−1^ for 10 min and the absorbance of the supernatant was measured at 541 nm by UV‒Vis spectroscopy (UV-2600i, SHIMADZU, Japan). The hemolysis ratio was calculated using Eq. (3):

3 $$Hemolysis\,(\% ) = \frac{{A_{s} - A_{{nc}} }}{{A_{{pc}} - A_{{nc}} }} \times 100\%$$

where *A*_*s*_, *A*_*pc*_, and *A*_*nc*_ are the absorbance values of the sample, positive control, and negative control, respectively.

#### Clinical Testing of Blood Samples from Liver Failure Patients

The study protocol was approved by the *Ethics Committee of Ruijin Hospital, Shanghai Jiao Tong University School of Medicine* (approval no. 2024–123). All procedures involving human blood were conducted in accordance with institutional guidelines, and written informed consent was obtained from every patient with hyperbilirubinemia.

*Preparation of Plasma* Plasma from ten patients with hyperbilirubinemia due to liver failure, cholestasis, or other related conditions (TBIL concentration ≥ 141 µmol L^−1^; see Table S8 for patient clinical characteristics) was collected via a plasma exchange-based artificial liver system, centrifuged at 4 °C, passed through a 0.45 µm filter, and stored at – 80 °C until use.

*TBIL and Alb Capture Experiments* After complete thawing, 5 mL of plasma per patient was transferred to a sterile Petri dish containing 60 mg of nano-HA-UiO-66 (pre-activated at 150 °C for 6 h) and circulated for 3 h in a shaker incubator (37 °C, 110 rpm). An equivalent mass of styrene–divinylbenzene-based anion exchange resin from a commercial IER disposable plasma bilirubin adsorption column served as the control. For each group, 150 μL of plasma was withdrawn at 0, 30, 60, 90, 120, 150, and 180 min, immediately centrifuged (10,000 rpm, 10 min) at 4 °C, and analyzed for TBIL and Alb levels with a clinical chemistry analyzer (SAL 9000, Mindray, China). The removal efficiencies (R, %) of TBIL and Alb were calculated using Eqs. (4) and (5) as follows:

4 $$R_{{TBIL}} \,(\% ) = \frac{{T_{0} - T_{e} }}{{T_{0} }} \times 100\%$$

5 $$R_{{Alb}} \,(\% ) = \frac{{C_{0} - C_{e} }}{{C_{0} }} \times 100\%$$

where *T*_*0*_ and *T*_*e*_ represent the initial and actual concentrations (μmol L^−1^) of TBIL, respectively, and *C*_*0*_ and *C*_*e*_ are the corresponding Alb concentrations (g L^−1^) in the samples.

### Statistical Analysis

All the data are presented as the means ± standard deviations of at least three independent replicates and were visualized with Origin Pro (2024b). T tests were used for comparisons between two groups, whereas one-way ANOVA was employed for comparisons among multiple groups. Unless otherwise specified, *p* < 0.05 was considered to indicate statistical significance.

## Results and Discussion

The nanocrystalline UiO-66 stoichiometry is Zr_6_O_4_(OH)_4_(BDC)_6_, in which Zr_6_O_4_(OH)_4_ clusters alternate with deprotonated terephthalate (BDC^2−^) to form a three-dimensional framework [43]. To exclude extrinsic interference and verify the coordination of the monodentate aliphatic carboxylate, a single protocol—ZrCl_4_/XA/H_2_BDC in *N*, *N*-dimethylformamide (DMF) at 120 °C—was adopted throughout. ZrCl_4_ served as the zirconium source, H_2_BDC served as the ditopic linker, and XA served as the capping modulator. XA ligands of increasing chain length and decreasing acidity—HA (p*K*a < 0), FA (p*K*a 3.77), BA (p*K*a 4.82), and OA (p*K*a 4.85)—were selected to systematically tune the defect density, pore size, and pore volume [44]. Specifically, excess capping agent is first premixed with ZrCl_4_ to saturate the surface of nascent Zr–O clusters with monocarboxylates, generating readily exchangeable secondary nanoporous building units. Sub-stoichiometric H_2_BDC was then introduced; its higher acidity (lower p*K*a) thermodynamically drives the quantitative displacement of XA and bridges adjacent Zr^4+^ ions, completing ligand exchange to yield the newly engineered UiO-66 nanoporous frameworks. The synergy between the XA chain length and acidity dictates the exchange efficiency: Longer chains increase the p*K*a and weaken the acid strength, amplifying the thermodynamic driving force for replacement but simultaneously introducing steric hindrance that kinetically suppresses further coordination. Consequently, increasing the XA alkyl chain length reduces the effective connectivity between Zr–O clusters and H_2_BDC. Upon activation (to completely remove XA), additional defects are generated, and the lattice is transformed into hierarchically porous architectures designated as nano-HA-UiO-66, FA-UiO-66, BA-UiO-66, and OA-UiO-66, which are collectively denoted as UiO-66 nanoporous frameworks.

### Morphology and Structural Characterization

#### ***TEM and ***^***1***^***H NMR Analysis***

As shown in Fig. 1, the newly engineered UiO-66 nanoporous frameworks are uniform, white powders (~ 200 nm; Figs. S1 and S2) with well-defined polyhedral morphologies (Fig. 1a). To eliminate spectral interference from residual DMF—which decomposes into formate and dimethylamine under basic or heated conditions—and to verify the complete removal of fatty acids after acid treatment, ^1^H NMR was employed to track chemical shift changes (Fig. 1b). Post-acidification spectra (Fig. 1c) reveal the complete disappearance of the formate resonance (δ = 8.06 ppm) for nano-HA- and FA-UiO-66, the butyrate signals (δ = 0.98, 1.68, 2.33 ppm) for nano-BA-UiO-66, and the octanoate signals (δ = 0.89, 1.29, 1.63, and 2.13 ppm) for nano-OA-UiO-66, confirming that the aliphatic monocarboxylic capping agents are fully removed and do not remain as compensatory ligands within the developed UiO-66 nanoporous frameworks.

#### FT-IR and XRD Analysis

**Fig. 2:**
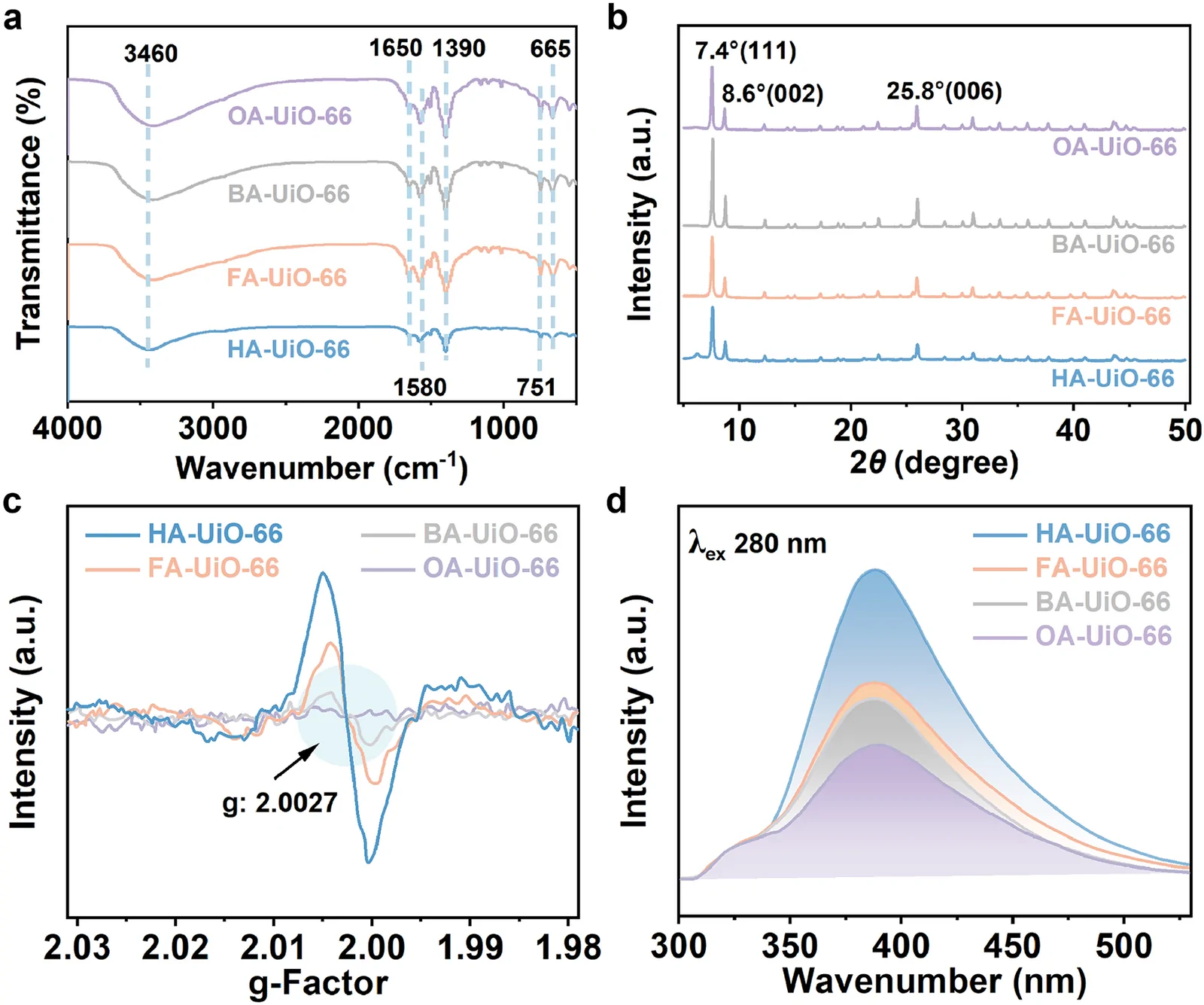
**a** FT-IR spectra,** b** XRD patterns, **c** EPR spectra, and **d** fluorescence emission spectra of the developed nano-HA-UiO-66, FA-UiO-66, BA-UiO-66, and OA-UiO-66

Fourier transform infrared spectroscopy (FT-IR) and X-ray diffraction (XRD) corroborate the chemical purity and crystallinity of the newly developed UiO-66 nanoporous frameworks. The characteristic Zr_6_ cluster vibrations at 900–1200 cm^−1^ and a marked attenuation of the -OH stretch (3460 cm^−1^) are shown in Fig. 2a, confirming effective XA removal [45]. New bands at 1452 and 1623 cm^−1^ indicate bilirubin confinement, whereas the C–O shift from 1650 to 1693 cm^−1^ and intense signals at 1150, 1076, 990, and 545 cm^−1^ identify -OH as the primary active site [46, 47]. Changes in phenyl C–C stretching (1650 ~ 1459 cm^−1^) further indicate that the aromatic ring is adsorbed. The crystalline structure of the developed UiO-66 was further verified by AC-TEM (Fig. S2a) and by Bragg reflections at 7.4°, 8.6°, and 25.8° indexed to the (111), (002), and (006) planes, respectively (Fig. 2b) [45], affirming that the phase-pure, highly nanocrystalline materials were suitable for subsequent investigations.

#### EPR and Fluorescence Spectral Analysis

Electron paramagnetic resonance (EPR) spectroscopy, together with fluorescence and absorption spectral changes, was used to resolve local structural differences among the engineered UiO-66 nanoporous frameworks and to identify putative binding sites for bilirubin. As shown in Fig. 2c, the EPR signal at g ≈ 2.0027 decreases monotonically with decreasing acid strength (HA to OA), indicating that longer alkyl chains hinder H_2_BDC coordination to adjacent Zr–O clusters and expose additional undercoordinated Zr^4+^ sites, thus increasing the defect density and confirming the proposed defect formation mechanism. The fluorescence spectral absorption intensity (Fig. 2d) corroborates the presence of these unsaturated centers in the engineered UiO-66 nanoporous frameworks [48, 49]: upon 280 nm excitation, ligand-to-metal (H_2_BDC to Zr–O cluster) charge transfer yields intense emission at 395 nm. Hence, the fluorescence signal quenching with increasing chain length of the newly developed nanoporous frameworks is ascribed to the reduced coordination number and diminished radiative recombination efficiency.

#### Thermodynamic Performance Analysis

**Fig. 3:**
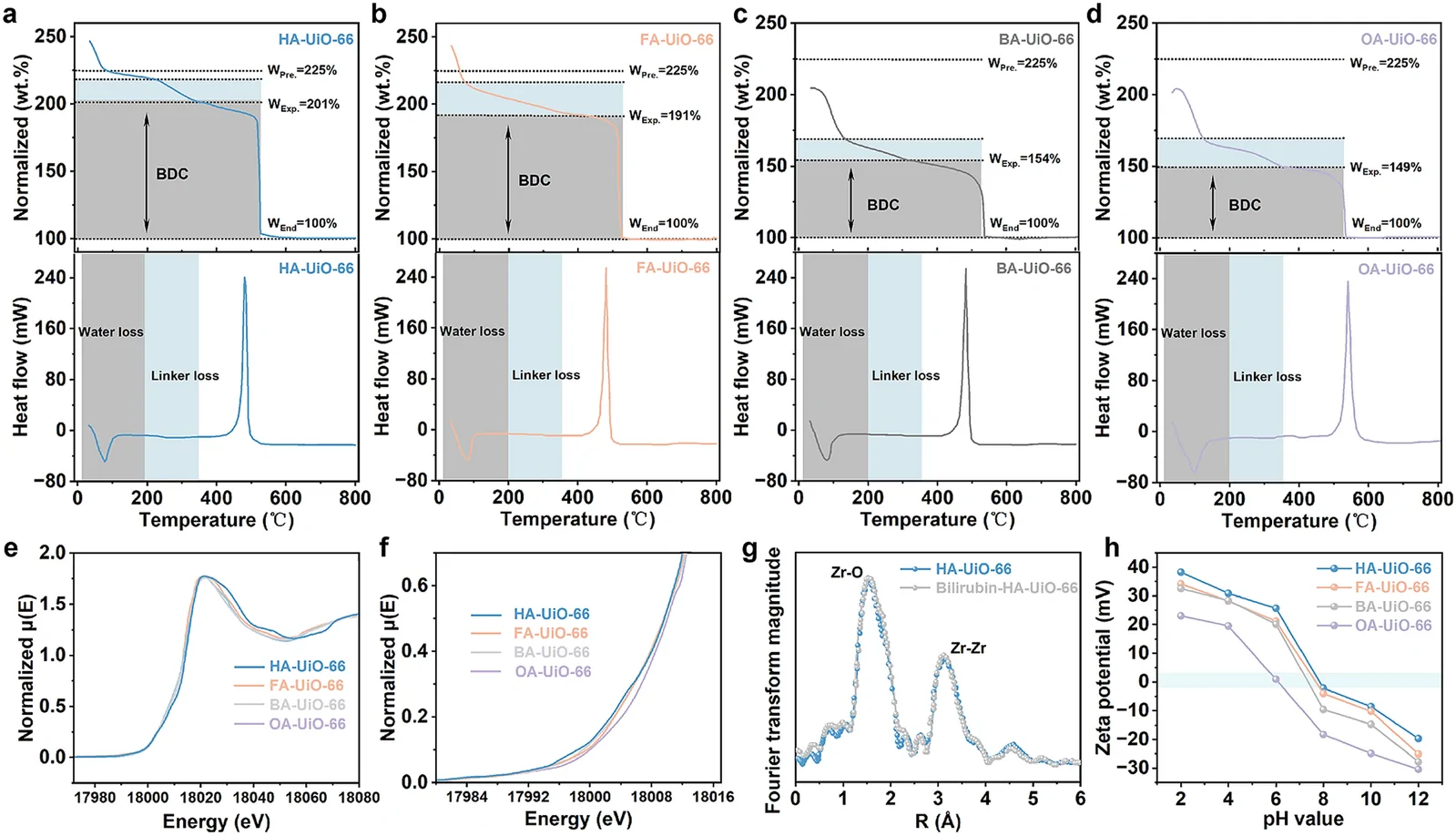
TGA‒DSC thermal analysis of **a** HA-UiO-66, **b** FA-UiO-66,** c** BA-UiO-66, and **d** OA-UiO-66 nanocrystals. **e** XAS spectra, **f** K-side XANES spectra,** g** EXAFS spectra, and** h** zeta potential versus pH of nano-HA-UiO-66, FA-UiO-66, BA-UiO-66, and OA-UiO-66

Simultaneous thermal analyses were performed in air to quantify the compositional fractions of the developed UiO-66 nanoporous frameworks. The normalized TGA traces (Fig. 3) reveal three distinct mass loss regions: (i) 30–100 °C, ascribed to the desorption of physisorbed water [50]; (ii) 200–350 °C, corresponding to the dehydroxylation of the metal nodes [51]; and (iii) 420–560 °C, associated with the collapse of the nanoporous framework, which was corroborated by exothermic peaks in the differential scanning calorimetry (DSC) profiles [51]. Mass losses in the 200–350 °C interval increase from 6% (nano-HA-UiO-66, Fig. 3a) to 7.6% (nano-FA-UiO-66, Fig. 3b), 9.2% (nano-BA-UiO-66, Fig. 3c), and 13% (nano-OA-UiO-66, Fig. 3d), yielding calculated Zr–O coordination numbers of 5.1, 4.7, 3.2, and 2.5 (Eqs. S1–S3), respectively. Therefore, the weaker acid strength of the monocarboxylate modulator leads to a systematically higher defect concentration in the developed UiO-66 nanoporous frameworks.

#### XAS and EXAFS Analysis

To elucidate the internal structural heterogeneity of the engineered UiO-66 nanoporous frameworks, the oxidation state and local coordination environment of Zr in the defect-engineered samples were probed by X-ray absorption spectroscopy. The Zr valence essentially remains unchanged after monocarboxylic acid modulation, yet a systematic blueshift of the Zr K-edge XANES is observed as the acid strength decreases (Fig. 3e, f). EXAFS (Fig. 3g) displays two dominant peaks at 1.7 and 3.1 Å, assigned to Zr–O and Zr–Zr scattering paths, respectively, indicating four distinct Zr–O contributions (μ_3_-O, μ_3_-OH, -COOH and -OH) within the first shell and accounting for the pronounced asymmetry in R-space [52]; the fitting parameters are summarized in Table S2. Coupled with the aforementioned characterization results and the zeta potential data (Fig. 3h), the positively charged surface of nano-HA-UiO-66 offers the most favorable electrostatic match for highly electronegative bilirubin, indicating that it has the highest capture performance.

### Adsorption Isotherms of the Engineered UiO-66 Nanoporous Frameworks

#### ***N***_***2***_*** Adsorption‒Desorption Isothermal Test***

**Fig. 4:**
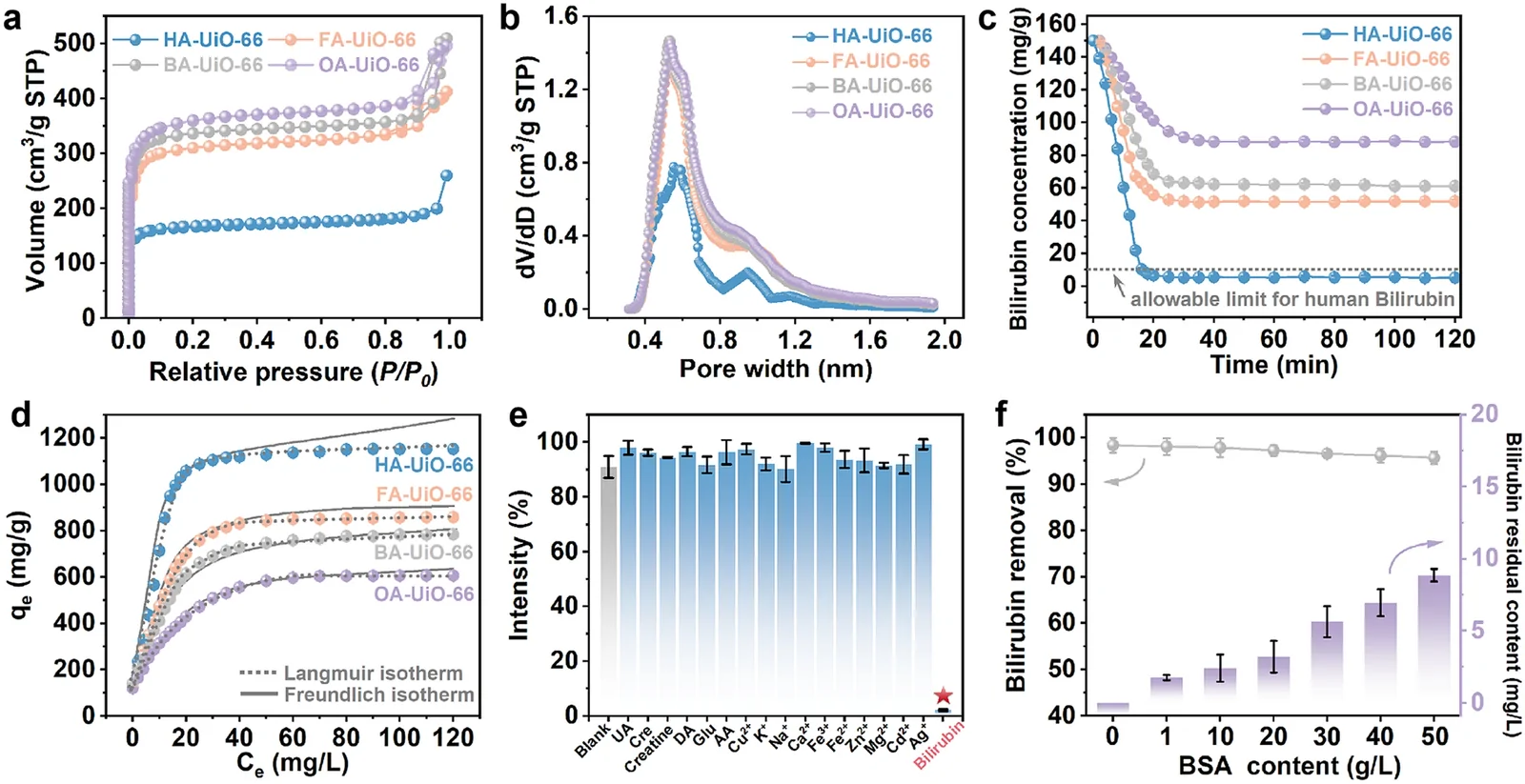
**a** N_2_ adsorption‒desorption isotherm curves (77 K) and **b** corresponding pore size distributions of the engineered UiO-66 nanoporous frameworks; **c** kinetic profiles of bilirubin (150 mg L^−1^) removal versus contact time for different engineered UiO-66 nanocrystals (0.8 g L^−1^); **d** equilibrium adsorption isotherms for bilirubin on the different engineered UiO-66 nanoporous frameworks (concentration: 0.25 g L^−1^);** e** bilirubin capture performance of nano-HA-UiO-66 in the presence of 5 × 10^−4^ mol L^−1^ free bilirubin together with 10^–3^ mol L^−1^ other various interfering chemicals. Biomolecules: UA, uric acid; Cre, creatinine; DA, dopamine; Glu, glucose; and AA, ascorbic acid; **f** residual concentration and removal efficiency of bilirubin (200 mg L^−1^) by nano-HA-UiO-66 (0.1 g L^−1^) with different concentrations of BSA (0–50 g L.^−1^)

N_2_ physisorption isotherms at 77 K (Fig. 4a) were collected to quantify the pore architecture of the UiO-66 nanoframeworks. Type I curves confirm hierarchical micro-/mesopores; the Brunauer‒Emmett‒Telle (BET) specific surface areas increase from 1130 m^2^ g^−1^ (nano-HA-UiO-66) to 1403 m^2^ g^−1^ (nano-OA-UiO-66) with increasing elongation of the monocarboxylate alkyl chain. Pore size distributions derived from density functional theory (DFT) calculations and BET analyses reveal that the average pore width expands from 7.6 Å to 3.41 nm, indicating a chain length-driven shift from Å-scale micropores to mesopores, which is consistent with literature trends [45–47, 52] (Fig. 4b, Table S3). Given the 6.7 Å linear span of bilirubin versus the > 6 nm hydrated diameter of Alb, the sub-nanometre windows of nano-HA-UiO-66 provide direct size exclusion selectivity for bilirubin while rejecting Alb. Post-capture BET data (Table S4) reveal a pronounced decrease in surface area, pore volume and mean pore size, corroborating successful guest accommodation within the engineered nanoporous frameworks.

#### Capture Performance of the Engineered UiO-66 Nanoporous Frameworks on TBIL in Simulated Blood

After the adsorption‒desorption evaluation was performed, the TBIL capture performance of the engineered UiO-66 nanoporous frameworks was assessed in PBS (pH 7.4) to simulate physiological conditions. The time-resolved TBIL concentration is shown in Fig. 4c. The concentration of TBIL in nano-HA-UiO-66 sharply decreases to 7.6 mg L^−1^ within 18 min (equilibrium, within the normal clinical range) and reaches 5.01 mg L^−1^ after 120 min, corresponding to a 97.9% removal efficiency. Isotherm data (Fig. 4d) were fitted with Langmuir and Freundlich models; the Langmuir equation yielded superior R^2^ values (Table S5), indicating that monolayer adsorption consists of (i) bilirubin binding to Zr–μ_3_-OH sites and (ii) surface self-aggregation driven by hydrogen bonding and hydrophobic interactions [53, 54]. Considering its maximum adsorption capacity, rapid equilibrium time, and favorable kinetics, compared with other adsorbents in this nanoporous framework series, nano-HA-UiO-66 demonstrates superior TBIL capture performance.

#### Specific Capture Performance of Nano-HA-UiO-66 on Bilirubin in Simulated Blood

As shown in Fig. 4e, a simulated blood cocktail containing urea, creatinine, creatine, dopamine, and glucose was used to benchmark the selective bilirubin capture of nano-HA-UiO-66. The developed HA-UiO-66 retains ~ 98% of bilirubin while adsorbing < 9.1% of any other solute, underscoring its exceptional molecular specificity. Graduated bovine serum albumin (BSA) solutions (0–50 g L^−1^) further revealed that the superior bilirubin capture performance is virtually independent of the BSA concentration: TBIL removal remains ≥ 95.67%, whereas BSA uptake is < 8.9% (Fig. 4f). After seven regeneration cycles, the TBIL capacity still exceeded 96.38%, and the RSD was ≤ 2.13% (Fig. S3a), furnishing direct evidence that nano-HA-UiO-66 still efficiently excludes BSA and exhibits good precision. Moreover, the seven-round cyclic elution process had no significant effect on the structural characteristics or adsorption capacity (RSD ≤ 4.19%, indicating a low degree of data dispersion) of HA-UiO-66 (Fig. S3b, c). Overall, compared with all the other engineered nanoporous frameworks, nano-HA-UiO-66 has comprehensive advantages in terms of size sieving, kinetics, selectivity, and recyclability.

### Interaction Forces Analysis

Kinetic and isothermal adsorption data have established that the internal architecture of nano-HA-UiO-66 provides a decisive advantage during bilirubin adsorption. To elucidate the underlying interaction forces, we combined ICP‒MS elemental analysis, FT‒IR and XPS spectroscopies, and DFT calculations to correlate the adsorption mechanism with the nanoscale ligand framework of nano-HA-UiO-66.

#### *ICP*‒*OES Analysis*

First, SEM‒EDS (Fig. S4) and ICP‒OES were employed to quantify the mass fractions of Zr, C, O, and N in nano-HA-UiO-66 after bilirubin capture; the results are summarized in Table S6. Using the ideal UiO-66 formula Zr_6_O_4_(OH)_4_(BDC)_6_, the average terephthalate connectivity per cluster is calculated to be only 2.4, which is well below that of the newly engineered UiO-66(Zr) (5.04) and the theoretical maximum (6). The literature [42, 51] reports a minimum connectivity of 3 (i.e., ≥ 6 organic linkers per Zr_6_ cluster) for fully intact UiO-66 nanoporous frameworks. Thus, nano-HA-UiO-66 concurrently exhibits cluster and linker defects, which are consistent with the pore size distributions. These undercoordinated Zr centers, stabilized by compensating ligands, generate Zr–OH sites that are believed to act as the primary active centers for TBIL adsorption.

#### FT-IR Analysis

**Fig. 5:**
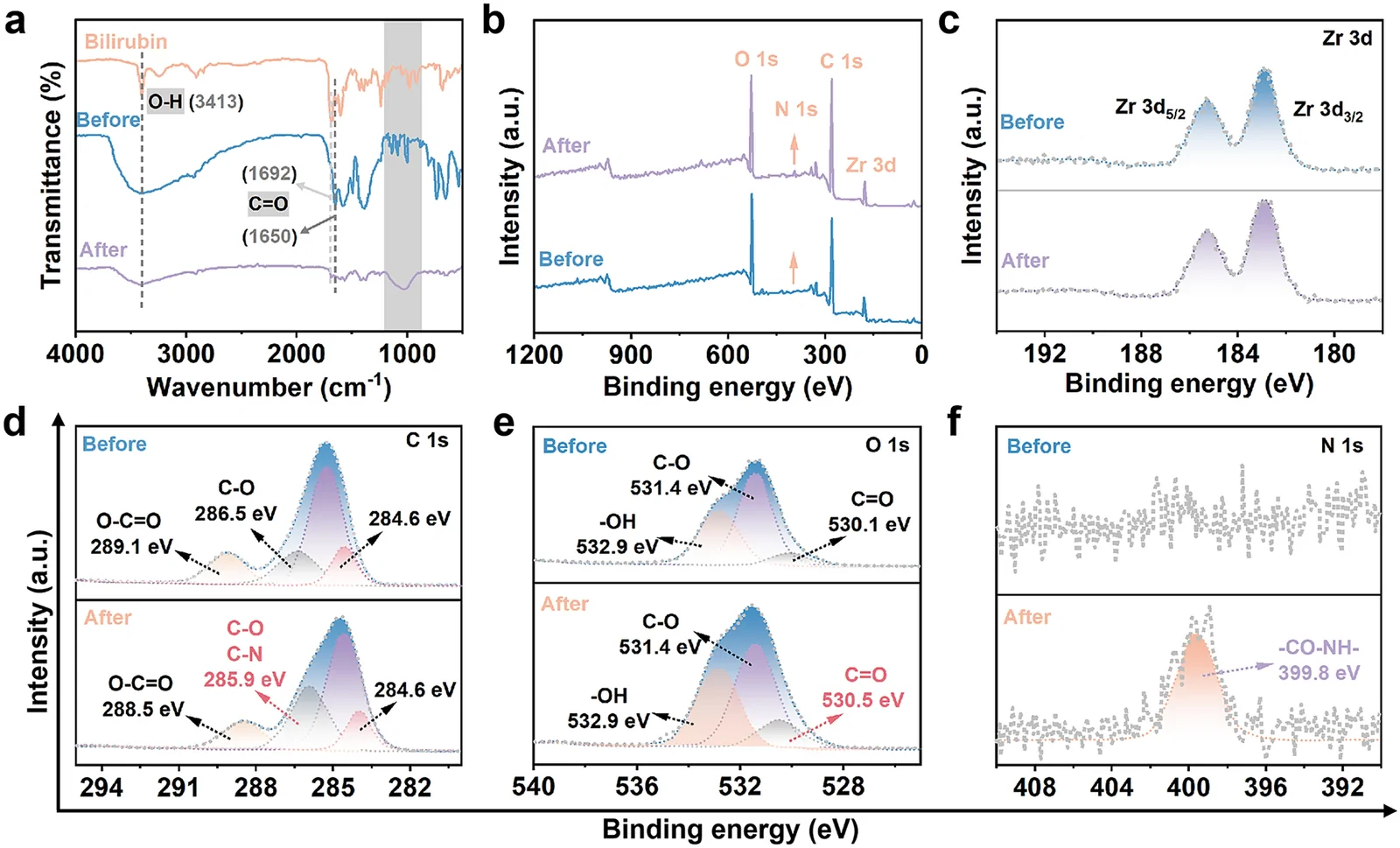
**a** FT-IR spectra and **b** XPS survey scans of nano-HA-UiO-66 before and after bilirubin adsorption. **c–f** High-resolution XPS regions:** c** Zr 3*d*, **d** C 1*s*, **e** O 1*s*, and **f** N 1*s*, respectively

The FT-IR spectra of nano-HA-UiO-66 before and after bilirubin capture are compared in Fig. 5a. New bands at 3413 cm⁻^1^ (O–H stretch of bilirubin), 1452 and 1623 cm⁻^1^ appear only after adsorption, confirming that bilirubin is entrapped within the engineered nanoporous framework [45, 55]. Concomitantly, the C–O stretching mode shifts from 1650 to 1692 cm⁻^1^, whereas intense, broad features emerge at 1150, 1076, and 990 cm⁻^1^. These changes indicate that bilirubin interacts specifically with Zr–OH sites (μ_3_–O, μ_3_–OH and Zr–O (–CO_2_)) [46], indicating a chemisorption mechanism; the pronounced modification of the μ_3_-OH vibration points to the strongest interaction. Additionally, the marked alteration of phenyl C–C stretching (1650 ~ 1459 cm⁻^1^) reveals π‒π stacking between the developed HA-UiO-66 nanoporous framework benzene rings and the bilirubin pyrrole moieties, constituting a further affinity site [46].

#### XPS Analysis

The results of the XPS analysis of nano-HA-UiO-66 before and after bilirubin capture are compared in Fig. 5b–e, with the elemental compositions and binding energies summarized in Table S7. After bilirubin capture, a distinct N 1*s* signal appears, accompanied by systematic blueshifts of the C–O and C = O components, confirming the insertion of the pyrrole moieties (C–N and C = O groups) of bilirubin into the engineered nanoporous framework of HA-UiO-66 [56], along with changes in surface zeta potential (Fig. S5). High-resolution spectra (Fig. 5c–f) reveal significant binding energy shifts for Zr–O, aromatic carbon, and carboxylate oxygen (C 1*s*: C–O from 286.5 to 285.9 eV; O–C = O from 289.1 to 288.5 eV; O 1*s*: C = O from 530.1 to 530.5 eV) [45]; The new N 1*s* feature peak at 399.8 eV, assigned to –CO–NH–, is consistent with the FT-IR trends, indicating that π-parallel stacking occurs between the bilirubin pyrrole rings and the BDC phenyl units as an additional affinity site. Notably, the Zr 3*d* (Fig. 5c) and N 1*s* (Fig. 5f) spectra indicate that, in the post-adsorption HA-UiO-66 sample, bilirubin does not directly coordinate with the coordination center of HA-UiO-66 via Zr–N bonding. Therefore, it can be inferred that the Zr–OH sites, which play a dominant role in bilirubin capture by HA-UiO-66, may originate from the interaction between coordinatively unsaturated Zr centers and compensating ligands (μ_3_-O/μ_3_-OH) in nano-HA-UiO-66.

#### DFT Simulation of Adsorption Models

**Fig. 6:**
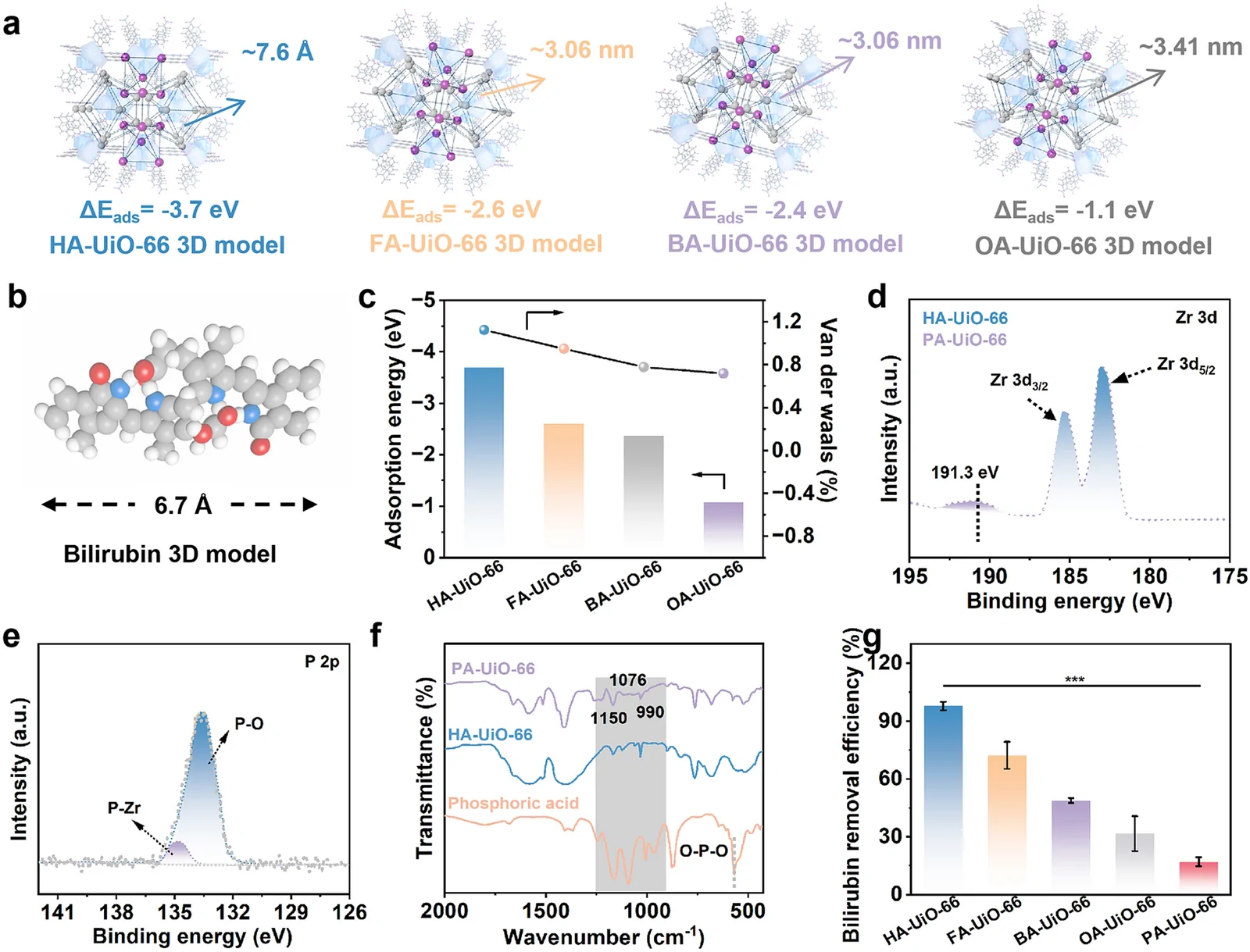
**a** DFT-calculated adsorption energies, pore size distributions and molecular simulations of the engineered UiO-66 nanoporous frameworks. **b** Ball-and-stick model of bilirubin (Mw = 584.67 g mol^−1^, molecular size 6.7 Å) generated in Materials Studio 8.0. **c** Relative contributions of van der Waals and adsorption energy to the total capture of bilirubin. High-resolution XPS regions of PA-UiO-66: **d** Zr 3*d* and **e** P 2*p*. **f** FT-IR spectra of phosphoric acid, original HA-UiO-66 and PA-UiO-66. **g** Bilirubin removal efficiency of HA-UiO-66, FA-UiO-66, BA-UiO-66, OA-UiO-66, and PA-UiO-66 under consistent initial bilirubin concentrations. (Data are expressed as the means ± SDs; ns = not significant; ***p < 0.001; n = 6)

To reconcile the DFT predictions with the experimental adsorption data while allowing bilirubin to enter the pores, periodic 3-D models of the developed UiO-66 nanoporous framework variants were constructed in Materials Studio 8.0 (Fig. 6a) [57]. Adsorption energies (ΔE_ads_) for bilirubin decrease along the series of nano-HA-UiO-66 (–3.7 eV) > FA-UiO-66 (–2.6 eV) > BA-UiO-66 (–2.4 eV) > OA-UiO-66 (–1.1 eV), confirming that the developed HA-UiO-66 nanoporous framework forms the strongest complex, which is consistent with a spontaneous, barrier-less capture event. Geometry analysis (Fig. 6b) revealed that the four N atoms of the bilirubin tetrapyrrole and the C atoms bridging the rigid pyrrole rings undergo the greatest conformational distortion inside the nano-HA-UiO-66 cavity, indicating a size-induced fit that stabilizes the guest. Furthermore, through simulation calculations, three cooperative hydrogen bonds between the carbonyl oxygens of bilirubin and the μ_3_–OH hydrogen atoms of the Zr_6_ node, augmented by van der Waals contacts, were identified; however, the latter contributed only ~ 1% to the total interaction energy (Fig. 6c). Thus, the results of DFT corroborate the previous experimental results that nano-HA-UiO-66 achieves superior bilirubin recognition through a combination of structural matching, H-bonding networking, and π–π stacking, whereas the aperture dimension (~ 7.6 Å) virtually eliminates Alb adsorption.

#### Functional Validation of Zr–μ₃-OH Sites

To validate the critical role of Zr–μ_3_-OH sites in bilirubin capture by HA-UiO-66, we performed competitive occupation experiments using phosphoric acid (PA) [23, 51, 58]. Specifically, HA-UiO-66 was soaked in a phosphate solution to allow phosphate ions to occupy the Zr–μ_3_-OH sites, yielding PA-UiO-66. In the Zr 3*d* XPS spectrum of PA-UiO-66, a distinct signal was observed at a binding energy of 191.3 eV (Fig. 6d), which is attributed to the interaction between the Zr atoms of HA-UiO-66 and the phosphate species. Moreover, the binding energy of P 2*p* in PA-UiO-66 blueshifted (133.8 eV) [52], providing direct evidence of chemical bonding between HA-UiO-66 and the adsorbed phosphate (Fig. 6e). Furthermore, successful phosphate coordination was confirmed by FT-IR (characteristic phosphate bands, Fig. 6f). As illustrated in Fig. 6g, compared with ~ 97% for pristine HA-UiO-66, PA-UiO-66 showed a drastically reduced bilirubin adsorption efficiency (~ 17%). Moreover, the HA-UiO-66 group exhibited an RSD of 2.04%, indicating good reproducibility. These results directly demonstrate that the Zr–μ_3_-OH sites play a decisive role in bilirubin capture, strongly supporting our proposed adsorption mechanism.

### Hemolysis Assay and Cell Compatibility of nano-HA-UiO-66

Hemocompatibility and cytocompatibility are fundamental prerequisites for any blood-contacting sorbent. Materials that induce hemolysis can disrupt erythrocyte membranes and release hemoglobin, thus compromising purification efficacy. To evaluate the hemocompatibility of nano-HA-UiO-66, we performed hemolysis assays using both rat and healthy human blood. As illustrated in Fig. S6a, b, the extent of hemolysis was quantified by measuring the absorbance of hemoglobin released from the supernatant at 541 (rat) and 545 nm (human). Even at the highest tested concentration (1,600 µg mL^−1^), the hemolysis ratios remained below 0.5% for rat blood and 0.8% for human blood, well within the 5% safety threshold for biomaterials. Furthermore, complement activation and coagulation effects were evaluated using specific ELISA kits and coagulation analyzers (Fig. S6c, d). Compared with the negative control, HA-UiO-66 did not significantly increase C3 or C5 levels, nor did it significantly prolong APTT, PT, or TT (*p* > 0.05, n = 6). These findings confirm that the material does not interfere with the intrinsic, extrinsic, or common coagulation pathways. Additionally, the cytocompatibility of nano-HA-UiO-66 was assessed using HEK293 and HUVEC cells. Following a 48-h incubation with various concentrations of HA-UiO-66 extracts, both cell lines maintained normal proliferation (Fig. S6e, f). Collectively, these results demonstrate that nano-HA-UiO-66 possesses excellent hemocompatibility and cytocompatibility, ensuring its safety for clinical hemoperfusion applications.

### TBIL Capture from Clinical Blood Samples by Nano-HA-UiO-66

**Fig. 7:**
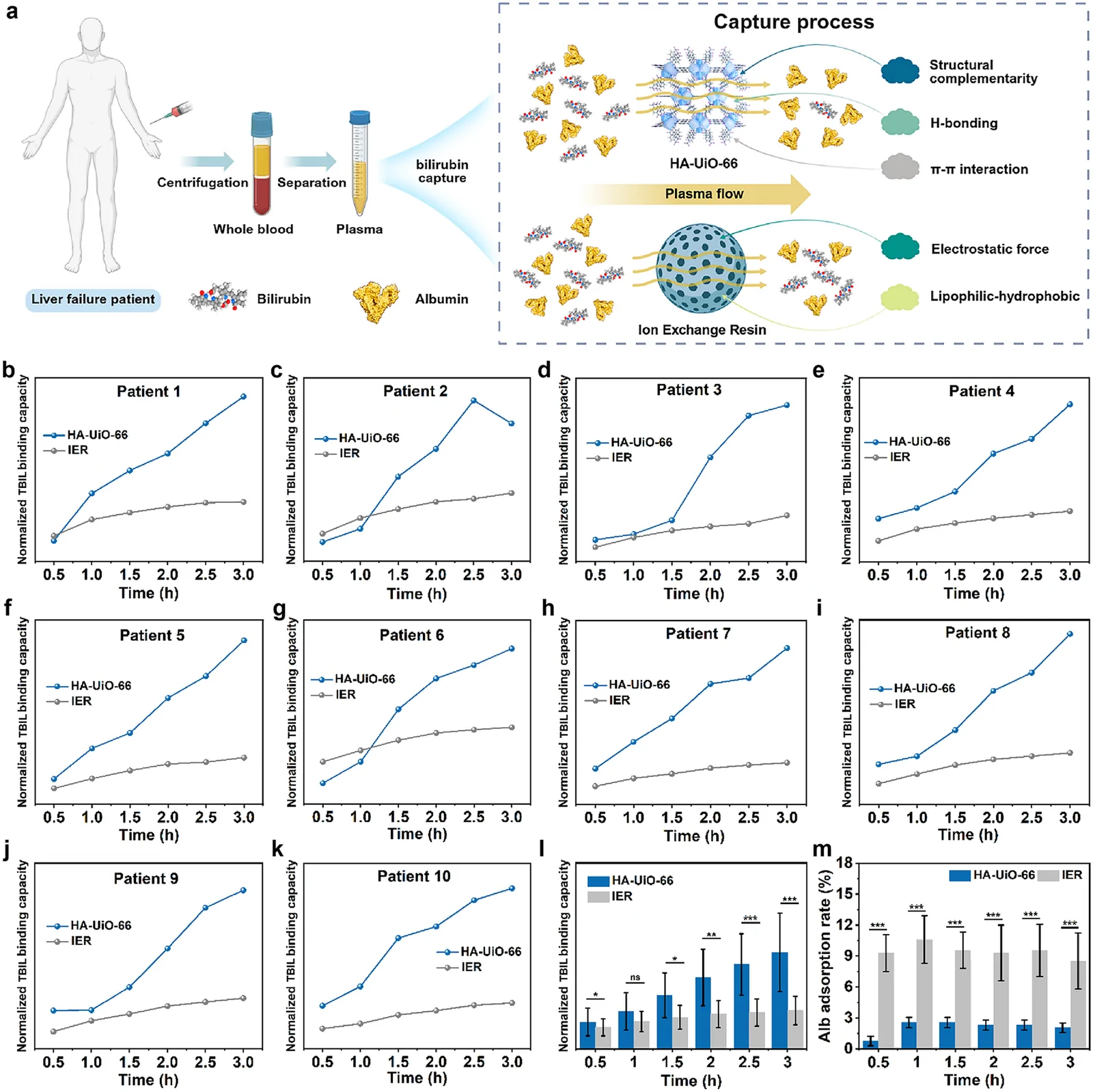
**a** Schematic of the clinical testing protocol (partially rendered with BioRender.com). **b–k** Time-resolved removal of TBIL (≥ 141 µmol L^−1^) from the plasma of ten patients within 3 h using nano-HA-UiO-66 or commercial ion exchange resin (IER, each column represents an individual patient). **l** Normalized TBIL capture capacity and **m** human Alb loss rate for nano-HA-UiO-66 versus commercial IER (Data are expressed as the means ± SDs; ns = not significant; *p < 0.05, **p < 0.01, ***p < 0.001; n = 10)

To evaluate the clinical translational potential of nano-HA-UiO-66, heparinized blood from ten patients [59–61] with hyperbilirubinemia due to liver failure, cholestasis, or other related conditions (TBIL ≥ 141 µmol L^−1^; see Table S8 for patient clinical characteristics) was processed in a single-pass, side-by-side comparison with a commercial disposable plasma bilirubin adsorption column, which uses styrene–divinylbenzene-based commercial IER as the primary active ingredient, as illustrated in Fig. 7a. The detailed statistical analysis results of the 10-patient datasets are presented in Table S9. The time-resolved TBIL profiles over 3 h are shown in Fig. 7b–k; the developed nano-HA-UiO-66 consistently exhibits faster kinetics and higher removal efficiency. Statistical analysis (Fig. 7l) revealed that nano-HA-UiO-66 consistently outperformed the commercial IER, demonstrating a remarkable 251% higher TBIL capture capacity at 3 h. After ≥ 1.5 h, compared with commercial IER, nano-HA-UiO-66 achieved a 100% superiority, with 70% of samples already exceeding the commercial baseline within the first hour (Fig. S7a). Crucially, nano–HA-UiO-66 maintained an exceptionally low Alb loss rate of ≤ 2.58% compared to ≥ 8.53% for the commercial IER (Figs. 7m and S7b–k), indicating that the lowest albumin leakage rate reported to date for clinical albumin-bound bilirubin clearance occurred (Fig. S7**l**). Although the intragroup RSD values for TBIL and Alb capture were relatively high (36.16% and 40.86%, respectively), they still met the acceptable standards for biological sample analysis, reflecting the significant biological variability and complex clinical conditions of hyperbilirubinemic patients [62–65]. These data comprehensively demonstrate the revolutionary clinical potential of nano-HA-UiO-66 in blood TBIL purification.

## Conclusion

In this study, by employing a monodentate–carboxylate ligand engineering strategy, we systematically tuned the coordination environment of UiO-66 nanoporous frameworks, generating hierarchical porosity and well-defined Zr–μ_3_-OH sites. Multiscale characterization, batch adsorption and DFT calculations reveal the structure‒property relationships governing bilirubin capture: the developed nano-HA-UiO-66 forms directional Zr pyrrole coordination with the bilirubin tetrapyrrole core, while sub-nanometer apertures physically exclude Alb. Additional hydrogen bonding and π‒π stacking further strengthen the complex. In clinical tests using plasma from ten liver failure patients, the TBIL removal capacity of nano-HA-UiO-66 was 251% greater than that of commercial IER, while Alb loss was maintained at ≤ 2.58%, breaking the long-standing trade-off between bilirubin clearance and essential protein depletion. Although the RSD values of clinical test data are relatively high (up to 40.86%), the complexity of biological sample characteristics in the medical field ensures that these data remain highly representative. These findings provide both mechanistic insight and a translational road map for the rational design of next-generation MOF nanocrystalline-based sorbents for selective blood purification.
